# ‘Muscular wisdom’ revisited: Decaying rates of stimulation mitigate torque loss

**DOI:** 10.1113/EP092472

**Published:** 2025-01-29

**Authors:** Raaj A. Dudani, Alexander M. Zero, Charles L. Rice

**Affiliations:** ^1^ School of Kinesiology, Faculty of Health Sciences The University of Western Ontario London Ontario Canada; ^2^ Department of Anatomy and Cell Biology, Schulich School of Medicine & Dentistry The University of Western Ontario London Ontario Canada

**Keywords:** contractile speed, electrical stimulation, high‐frequency fatigue, muscle fatigue, M‐wave, task dependence

## Abstract

During a sustained high‐intensity isometric maximal voluntary contraction (MVC), declining motor unit firing rates (MUFRs) accompany torque loss. This decline (∼50% over 60 s) helps to maintain torque by preserving peripheral electrical propagation and matching the slowing contractile properties with torque loss (i.e., ‘muscular wisdom’). However, it has been suggested that reduced MUFRs contribute to torque loss. Here, we compared torque loss between constant and decaying rates of electrical stimulation to mimic MUFRs reported during MVCs. The dorsiflexors of 8 males and 5 females (21–30 years) underwent three 60 s muscle fatiguing conditions: (1) sustained MVC; (2) constant high‐frequency electrical stimulation (40 Hz); and (3) exponentially decaying stimulation rate (from 40 to 20 Hz). The decaying rate demonstrated less torque loss compared with the sustained high‐frequency stimulation and the MVC conditions (*P* < 0.01). Furthermore, torque increased (by ∼17%, *P* < 0.005) when the constant high‐frequency condition was switched to 20 Hz for 2 s at task termination. Conversely, torque loss was accelerated when the decaying stimulation rate was switched from 20 to 40 Hz for 2 s at task termination (by ∼16%, *P* < 0.001). Following all conditions, evoked twitch responses slowed (by 29%–77%, *P* < 0.01) but M‐wave amplitude was reduced only for the constant high‐frequency condition (by ∼23%, *P* < 0.01). Thus, the reduction in stimulation rates maintained optimal activation by matching the fatigue‐induced contractile slowing in combination with preserved peripheral electrical conductance. Therefore, reducing the activation rate preserves torque, rather than contributing to torque loss during high‐intensity contractions, thereby supporting the muscle wisdom hypothesis.

## Abstract (highlights)

- **What is the central question of the study?**
  We aimed to resolve the discrepancy between studies using electrical stimulation to evaluate the ‘muscular wisdom’ hypothesis, which suggests that decaying motor unit firing rates (MUFRs) during sustained maximal voluntary isometric contractions (MVC) minimize torque loss.
- **What is the main finding and its importance?**
  Decaying rates of electrical stimulation produced less torque loss than constant high rates. Both conditions produced contractile slowing, suggesting that lower rates of activation were adequate for torque maintenance. Constant high rates reduced M‐wave size, whereas decaying rates did not. Therefore, the commonly reported decline in MUFRs during sustained MVCs helps to mitigate torque loss by preserving electrical propagation and matching fatigue‐induced contractile slowing.

## 1 INTRODUCTION

Conventionally, muscle fatigue can be characterized by a reduction in the ability to generate torque or force (Bigland‐Ritchie et al., 1995; Taylor et al., 2016). During an isometric maximal voluntary contraction (MVC), the loss in contractile generating capacity is accompanied by a decline in motor unit firing rates (MUFRs), which can be as much as 50% from initial rates during a 60 s contraction (Bigland‐Ritchie et al., 1983; Grimby & Hannerz, 1977; Marsden et al., 1983; cf. Macefield et al., 2000). Historically, it was suggested that the reduction in MUFRs is a compensatory mechanism to minimize torque loss (Bigland‐Ritchie & Woods, 1984; Bigland‐Ritchie et al., 1979; Jones et al., 1979; Marsden et al., 1976), termed ‘muscular wisdom’ (Marsden et al., 1983); however, this principle was re‐evaluated several years later to address some important limitations identified in the original studies and concluding that the reduction in firing rates are a contributor to torque loss (i.e., absence of muscle wisdom) (Fuglevand & Keen, 2003). We have identified that each approach to this overall observation has limitations that preclude a definitive conclusion on whether a change in motor unit output contributes to contractile impairment (i.e., muscle fatigue) or whether it helps to mitigate a decline in contractile capacity. The present study was designed to address the main limitations of these contrasting findings, thereby reassessing the muscle wisdom hypothesis.

Evidence to support muscle wisdom is derived primarily from comparing voluntary and electrically stimulated contractions. During sustained 60 s stimulation at 80 Hz, force loss was greater than a sustained MVC. Indeed, to match the fatigability (i.e., force loss) of an MVC, electrical stimulation with declining rates of activation (e.g., from 60 to 20 Hz) was required (Jones et al., 1979). Furthermore, in a separate condition, when sustained stimulation of 80 Hz was interrupted with 20 Hz, the force output was improved ∼30 s into the contraction, despite the 20 Hz initially producing less force (Jones et al., 1979). Therefore, a reduction in activation rate seemed beneficial in minimizing force loss, and the lower rates of activation improved force output during acute muscle fatigue. This and similar findings by another group (Marsden et al., 1983) with electrically stimulated contractions prompted the muscle wisdom hypothesis. It was argued that torque loss was minimized because declining MUFRs during maximal contractions maintained optimal activation by matching the fatigue‐induced muscle contractile slowing and limiting potential impairments of peripheral conduction to the muscle (Bigland‐Ritchie & Woods, 1984; Bigland‐Ritchie et al., 1979; Jones et al., 1979; Marsden et al., 1983).

Throughout the course of a sustained MVC, in addition to a loss of force, contractile speed slows, as evidenced by longer twitch relaxation times and a leftward shift in the electrically stimulated force–frequency relationship (Bigland‐Ritchie et al., 1983). As a result of the slowing in contractile kinetics, force summation is improved during fatigue and thus tetanic (fused) contractions can be maintained with lower MUFRs (Bigland‐Ritchie & Woods, 1984; Bigland‐Ritchie et al., 1983). For example, following a 1 min MVC, summation of unfused subtetanic stimulated (e.g., 10 Hz) forces are improved, becoming a greater fraction of maximal tetanic force (Bigland‐Ritchie & Woods, 1984; Bigland‐Ritchie et al., 1983). Therefore, despite the reduction in MUFRs, adequate muscle activation might be maintained better by taking advantage of the fatigue‐induced contractile slowing and improved force summation.

The decline in MUFRs during a sustained MVC also minimizes impairments in peripheral conduction (Bigland‐Ritchie et al., 1982). In response to a single electrical pulse, the M‐wave represents the summation of motor unit action potentials within the vicinity of the electromyographic (EMG) electrode and can be used to assess peripheral conductivity in studies of muscle fatigue (Bigland‐Ritchie et al., 1979; Merton, 1954; Thomas et al., 1989). During sustained MVCs, in which maximal MUFRs decline (Bigland‐Ritchie et al., 1982; Grimby & Hannerz, 1977; Marsden et al., 1983; cf. Macefield et al., 2000), M‐wave amplitude is well maintained (Bigland‐Ritchie et al., 1979, 1982; Merton, 1954). However, when the muscle is activated with sustained high rates of electrical stimulation (e.g., 60 Hz) there is a decline in M‐wave amplitude in addition to the greater loss of force in comparison to a sustained MVC (Bigland‐Ritchie et al., 1979; Jones et al., 1979; Marsden et al., 1983). Furthermore, when initially high rates of electrical stimulation are reduced (e.g., from 60 to 20 Hz) during a sustained stimulated contraction, the M‐wave amplitude is maintained, and force loss matches a sustained MVC (Bigland‐Ritchie et al., 1979; Jones et al., 1979; Marsden et al., 1983). Therefore, the decline in maximal MUFRs could minimize force loss by maintaining peripheral electrical propagation.

More recently, experimental evidence was provided that did not support the muscle wisdom hypothesis (Fuglevand & Keen, 2003). At the time of the original experiments, less was known about maximal MUFRs, and indeed, the maximal MUFR of the adductor pollicis is ∼30 Hz (Bellemare et al., 1983), well below the 80 and 60 Hz stimulations used (Bigland‐Ritchie et al., 1979; Jones et al., 1979; Marsden et al., 1983). Therefore, with a similar experimental protocol but using ‘physiological’ maximal rates (i.e., 30 Hz), Fuglevand and Keen (2003) showed that force was better maintained if a high stimulation rate was kept constant compared with both a sustained MVC and declining rates of stimulation (i.e., from 30 to 15 Hz). Those results indicated that declining rates of activation would be a causative factor in force loss and not preventive. Since that study, this has been a well‐accepted viewpoint of muscle fatigue (e.g., Barry & Enoka, 2007; Taylor & Gandevia, 2008).

Besides the rates of stimulation, a key difference between these primary studies was the stimulated force output. The original studies (Bigland‐Ritchie et al., 1979; Jones et al., 1979; Marsden et al., 1983) used unphysiologically high rates of activation, because these were required to induce near MVC force with electrical stimulation. However, the physiological rates used by Fuglevand and Keen (2003) evoked only ∼55% MVC force. It is well appreciated that the mechanisms of force loss are highly task dependent (Bigland‐Ritchie et al., 1995; Taylor & Gandevia, 2008). For example, during sustained submaximal contractions at 50% MVC, MUFRs can increase to compensate for impaired torque generation (Carpentier et al., 2001; Valenčič et al., 2024), which does not occur during maximal fatiguing contractions (Bigland‐Ritchie et al., 1982; Grimby & Hannerz, 1977; Marsden et al., 1983). Therefore, perhaps it is not surprising that maintaining a high rate of stimulation preserved force output in the experiments by Fuglevand and Keen (2003), because rate coding is a key mechanism to limit force loss in the submaximal task (i.e., ∼50% MVC) used in that investigation.

The purpose here was to assess prior limitations by using physiological rates of stimulation in a muscle group (dorsiflexors) capable of producing high‐intensity forces (e.g., 80% MVC) from electrical stimulation (Smith et al., 2014) and therefore similar to voluntary contractions and in a muscle group known to reduce maximal MUFRs during high‐intensity contractions (Zero et al., 2023). We hypothesized that the constant high‐frequency condition would yield the greatest force loss, whereas the MVC and decaying frequency conditions would result in lesser force losses. Furthermore, we hypothesized that prolonged (i.e., 30 min) recovery assessments would be more impaired for the MVC and decaying frequency conditions owing to their greater torque–time integrals resulting in greater peripheral impairments.

## 2 MATERIALS AND METHODS

### 2.1 Ethical approval

Participants provided oral and written consent before experimental testing, in accordance with the *Declaration of Helsinki*. Ethical approval was also provided by the local University's Review Board for Health Science Research Involving Human Participation (no. 107505).

### 2.2 Participants

Eight males (24.5 ± 2.7 years of age, 180.4 ± 7.7 cm tall and weighing 82.1 ± 11.1 kg) and five females (21.4 ± 0.5 years of age, 169.5 ± 4.9 cm tall and weighing 67.1 ± 8.4 kg) volunteered to participate in the study (*n* = 13). Using verbal screening, participants were free of neuromuscular and metabolic disorders. All participants were instructed to avoid strenuous lower‐limb exercise for 24 h before each testing session.

### 2.3 Experimental arrangement

Participants were seated upright on a chair with their non‐dominant leg placed in a custom‐made torque dynamometer to record isometric dorsiflexion contractions (Marsh et al., 1981). The hip and knee joints were placed at 90°, while the ankle joint was placed in 10° of plantar flexion to minimize contributions from the fibular muscles (Marsh et al., 1981). Two in‐elastic straps were tightly secured over the dorsum of the foot to the dynamometer, in addition to a C‐clamp that was placed over the distal anterior thigh to minimize knee and hip movements during the contractions. Torque was transmitted through a rigid footplate that had a strain gauge situated at the axis of ankle joint rotation. The signal was converted from analog to digital (Power1401, Cambridge Electronic Design, Cambridge, UK) and was sampled at 500 Hz (Spike2, Cambridge Electronic Design). A monitor was placed in the visual field of the participants, ∼1 m away, to provide real‐time feedback of their torque responses.

### 2.4 Fibular nerve stimulation

To produce electrically stimulated dorsiflexion contractions, a bipolar bar electrode coated with electrode gel was held firmly in place over the skin by the experimenter, posterior and inferior to the head of the fibula, over the common fibular nerve. Using a single pulse, stimulation intensity (DS7AH, Digitimer Ltd, Welwyn Garden City, UK) was increased incrementally until the amplitude of muscle twitch torque and M‐wave peak‐to‐peak amplitude reached a peak that was unchanged despite an increase in current. Current intensity was then increased by an additional ~20% to ensure supramaximal stimulation throughout the experiment. To produce high‐frequency stimulated contractions, 40 Hz was used as the mean upper physiological range of tibialis anterior (TA) firing rates, as previously reported from brief MVCs (Connelly et al., 1999). The same current intensity obtained from maximal twitch and M‐wave responses was used for all 10 and 40 Hz stimulated contractions. Square‐wave pulses for stimulation had a duration of 200 µs, and the stimulator was set at maximum voltage (400 V). The stimulation intensity for participants ranged from 14.4 to 20.8 mA for all electrically evoked contractions.

### 2.5 Electromyography

Surface EMG was recorded from the TA muscle in a monopolar arrangement using self‐adhering electrodes (ECG electrodes, GE Healthcare). The active electrode was placed over the TA muscle belly at the lower third of the tibia (Thomas et al., 1989), and the reference and earth electrodes were placed on the lateral malleolus. Surface EMG signals were preamplified (×100), filtered between 5 Hz and 10 kHz (Neurolog, NL844, Digitimer, Welwyn Garden City, UK), and sampled at 2500 kHz (Spike2, Cambridge Electronic Design). Before electrode placement, the recording sites were cleaned with 70% ethanol. During determination of supramaximal stimulation, the active electrode was repositioned (∼2 cm) along the muscle belly to minimize M‐wave rise time, ensuring that electrode placement was close to the motor point (Power et al., 2010).

### 2.6 Familiarization

All participants underwent a familiarization session prior to the three testing sessions that occurred on separate days spaced ≥72 h apart. Participants practised performing sustained MVCs (5–30 s) and were familiarized with brief supramaximal 40 Hz stimulations each for 2–3 s.

### 2.7 Fatiguing conditions

Among the three experimental sessions, representing the three fatiguing conditions, the experimental procedures were equivalent except for the 60 s fatiguing condition that was selected at random. The three 60 s fatiguing conditions were as follows: (1) constant high‐frequency 40 Hz stimulation rate; (2) exponentially decaying stimulation rates from 40 to 20 Hz; and (3) a sustained MVC.

Immediately after 60 s of the 40 Hz stimulation condition, the stimulation frequency was switched to 20 Hz for an additional 2 s to compare the effect of lower frequencies on torque production. For the exponentially decaying stimulation condition, rates began at 40 Hz but were lessened to 20 Hz at an exponential constant of 1.155 over the 60 s. These parameters were selected to closely represent physiological rates of decay in MUFRs of the TA reported previously during a 60 s dorsiflexion MVC (Zero et al., 2023). Immediately after 60 s of decaying stimulation, the 20 Hz was switched to 40 Hz for 2 s to compare the effect of higher frequencies on torque production at this stage.

The third condition consisted of a sustained 60 s MVC with supramaximal single pulses delivered at 2, 5, 10, 20, 30, 40 and 55 s to elicit and record M‐waves and to induce superimposed twitches used for the calculation of voluntary activation (VA; assessed only at 55 s) during the fatiguing task.

### 2.8 Experimental protocol

Supramaximal twitch and maximal 40 Hz currents were obtained as described above (see section 2.4). Isometric VA of the dorsiflexors was assessed before each fatiguing condition using the interpolated twitch technique (Todd et al., 2004). A single supramaximal stimulus was applied to the fibular nerve with the muscle at rest, during the torque plateau of a ∼3 s dorsiflexion MVC, and ∼2 s after the MVC with the muscle at rest. During dorsiflexion MVC attempts, participants were provided with strong verbal encouragement and real‐time visual feedback of their torque recordings.

**FIGURE 1:**
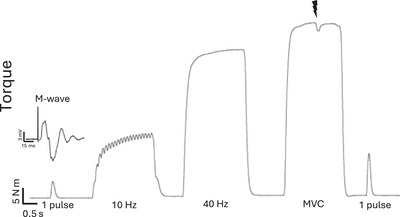
Unprocessed exemplar torque data (in newton metres, Nm) of a baseline sequence consisting of a brief electrically stimulated single pulse (i.e., muscle twitch), 10 Hz (2 s) and 40 Hz (2 s) contractions, followed by an interpolated twitch and a post‐MVC twitch. An unprocessed exemplar M‐wave of the tibialis anterior in response to a single pulse of electrical stimulation is provided above the evoked twitch response. The lightning bolt represents a superimposed twitch during MVC. Abbreviation: MVC, maximal voluntary contraction.

For all the three experimental sessions, a baseline sequence was performed, which consisted of brief electrical stimulation and voluntary contractions sequentially of a single pulse (i.e., muscle twitch), 10 Hz (2 s) and 40 Hz (2 s), followed by an interpolated twitch technique (Figure 1). This protocol was performed twice, with a minimum of 5 min of rest between sequences. Next, the specific fatiguing condition was assigned randomly (see section 2.7).

Immediately after the termination of each fatiguing condition, the interpolated twitch technique was used to assess voluntary activation and twitch contractile properties. To assess recovery of maximal and submaximal torque, the sequence as described above was repeated (Figure 1) at 2, 5, 10, 20 and 30 min after task failure.

### 2.9 Data analysis

All data analysis was performed offline using the software Spike2. For all MVCs, peak values were obtained by calculating the average torque response within ±0.5 s around the instantaneous point of peak torque. Torque at the termination of the fatiguing condition was obtained by averaging over the last 0.5 s (i.e., 59.5–60 s). For evoked twitches, 10 and 40 Hz stimulations, peak torque was defined as the maximal value in newton metres (Nm).

**FIGURE 2:**
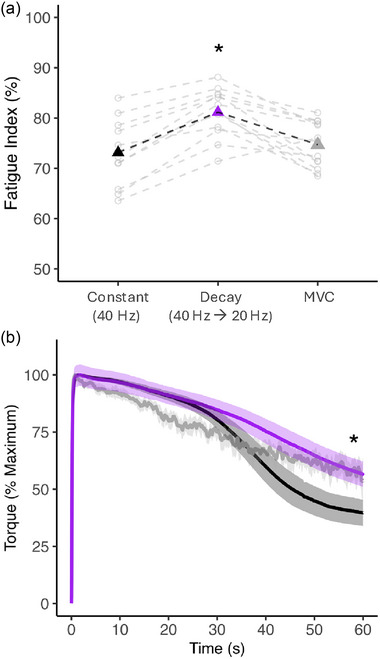
(a) Normalized fatigue indices for each condition and participant. (b) Mean torque traces (as a percentage of maximum) for each fatiguing condition. The black triangle (in a) and torque trace (in b) represent the constant high‐frequency condition. The purple triangle (in a) and torque trace (in b) represent the decaying stimulation condition. The grey triangle (in a) and torque trace (in b) represent the MVC condition. Shaded regions represent the 95% confidence interval. *The fatigue index of the decay condition (purple, 81.2% ± 4.7%) is significantly different from 40 Hz (black, 73.1% ± 6.1%) and MVC (grey, 74.7% ± 4.5%) conditions. Abbreviation: MVC, maximal voluntary contraction.

**FIGURE 3:**
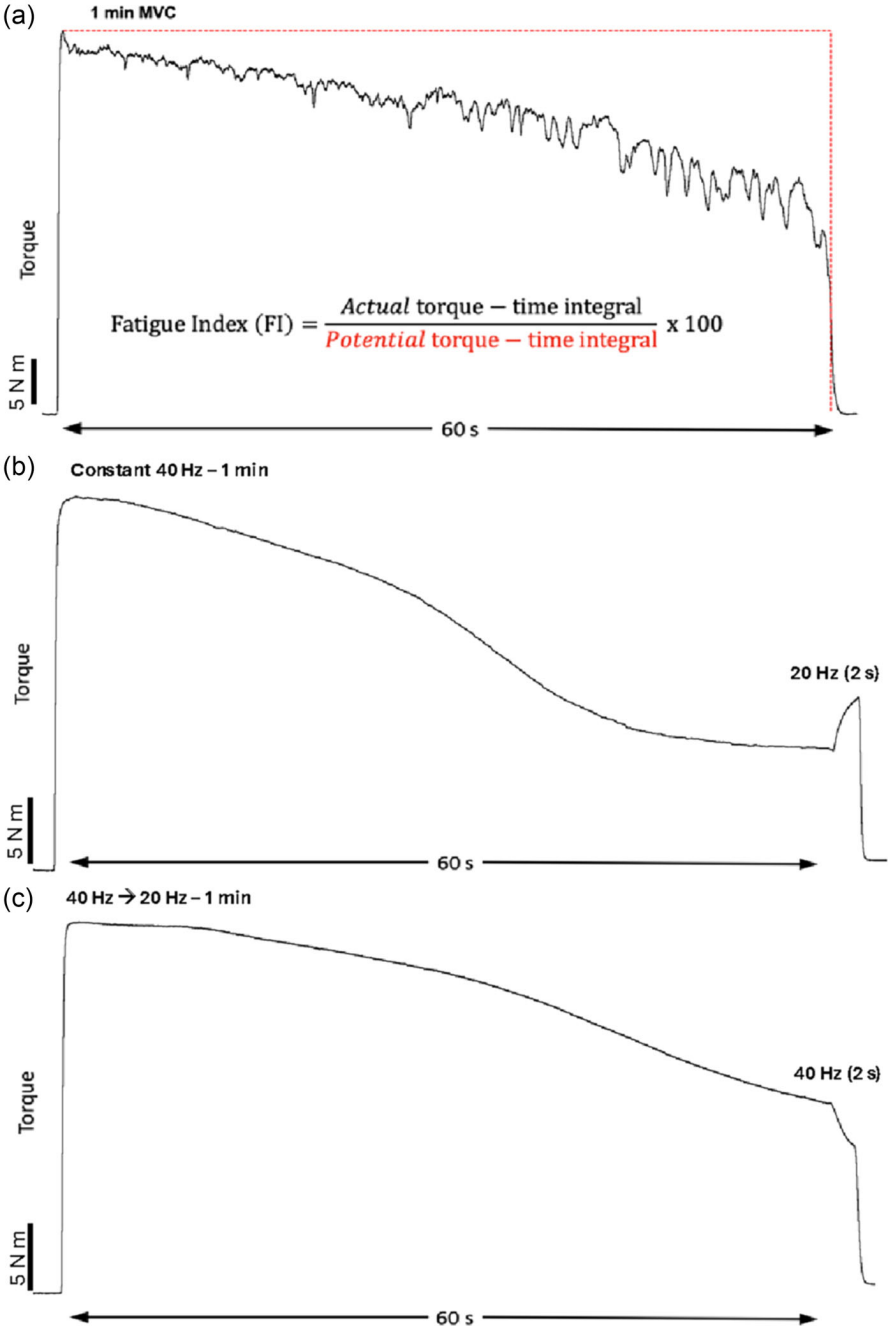
Exemplar unprocessed torque data (in newton metres, Nm) of each fatiguing condition. (a) Unprocessed torque trace of a participant performing the 60 s MVC. The equation used to calculate the normalized fatigue indices for each participant and condition is provided under the torque trace. (b) Unprocessed torque trace of a participant being stimulated at 40 Hz for 60 s, then at 20 Hz (60–62 s) to demonstrate the effect of physiologically high constant rates of activation on torque loss. (c) Unprocessed torque trace of a participant being stimulated by the declining stimulation condition from 40 to 20 Hz over 60 s at an exponential constant of 1.155, then at 40 Hz for an additional 2 s to demonstrate the effect of decaying rates of activation on torque.

For each fatiguing condition, a normalized fatigue index was calculated by obtaining the torque–time integral (area under the torque trace) and dividing it by the potential torque–time integral (peak torque multiplied by 60 s, representing no torque loss). These values were then converted into a percentage (see Figure 2 and 3). For the two electrical stimulation conditions (constant 40 Hz, and decreasing from 40 to 20 Hz), the torque change during the additional 2 s (i.e., 60–62 s) was calculated by taking the difference between the torque at the end of the 60 s fatiguing contraction and the torque at the end of the 2 s when the frequency was changed (Figure 3).

For each recovery sequence at 2, 5, 10, 20 and 30 min after contraction termination, the 10 Hz:40 Hz ratios were compared with baseline ratios to assess the presence of prolonged low‐frequency force depression. To reduce redundancies and the volume of data presented, only comparisons between 30 min after contraction termination and baseline are reported.

The M‐waves produced from all single pulses at rest and during MVCs were analysed to obtain parameters of area, peak‐to‐peak amplitude, duration and peak‐to‐peak duration. The M‐wave area was obtained between the stimulation onset and positive and negative inflections. Peak‐to‐peak amplitude was measured as the difference in voltage between the largest positive and negative inflections. Duration was measured as the time between the stimulation onset and the end of inflections, and peak‐to‐peak duration was measured as the time elapsed between positive and negative inflection peaks.

### 2.10 Statistical analysis

All statistical analysis was performed in R (v.4.1.2; R Foundation for Statistical Computing, Vienna, Austria). A one‐way repeated‐measures ANOVA was conducted to assess the effect of the fatigue condition (i.e., 40 Hz, MVC and 40 → 20 Hz) for the fatigue index. Two‐way repeated‐measures ANOVAs were performed to compare conditions (MVC, 40 Hz and 40 → 20 Hz) and time for measures of VA and M‐wave parameters. Student's paired *t*‐tests were used to compare twitch parameters and the 10 Hz:40 Hz ratio between conditions at baseline, task termination and 30 min after task termination. Student's paired *t*‐test was used to compare VA at 55 s during the sustained MVC to baseline. Student's paired *t*‐tests were used to compare the absolute torque and the rate of torque loss at the end of the 60 s electrically stimulated contractions and the torque when the frequency was switched for 2 s. For significant main effects and interactions, *post hoc* testing was conducted using Student's paired *t*‐tests with Holm–Šídák corrections. Data are presented as mean values ± SD, and α was set at *P* ≤ 0.05.

## 3 RESULTS

### 3.1 Baseline measurements

For the three fatigue conditions, baseline values of MVC torque, twitch torque, 10 Hz and 40 Hz torques, M‐wave parameters, twitch parameters and VA are reported in Table 1. Exemplar data of a baseline sequence are presented in Figure 1.

**TABLE 1:** Baseline measurements.

Parameter	Condition 1 (40 Hz)	Condition 2 (40 → 20 Hz)	Condition 3 (MVC)
Voluntary activation (%)	99.3 ± 0.6	98.9 ± 2.8	99.3 ± 1.3
MVC torque (N m)	40.3 ± 12.3	39.9 ± 12.1	40.3 ± 11.8
Twitch torque (N m)	3.3 ± 1.4	3.3 ± 1.6	3.6 ± 1.7
10 Hz torque (N m)	11.9 ± 5.7	11.4 ± 6.1	12.5 ± 6.1
40 Hz torque (N m)	33.3 ± 10.7 (83.5% ± 7.6%)	32.7 ± 10.9 (83.1% ± 7.4%)	32.4 ± 10.4 (81.6% ± 8.4%)
Tw:40 Hz	0.10 ± 0.02	0.10 ± 0.02	0.11 ± 0.03
10 Hz:40 Hz	0.35 ± 0.10	0.34 ± 0.11	0.38 ± 0.11
PTP amplitude (mV)	6.3 ± 2.5	7.0 ± 2.2	6.6 ± 1.9
Area (mV s)	56.0 ± 26.9	67.4 ± 25.7	69.1 ± 22.1
Duration (s)	0.032 ± 0.006	0.031 ± 0.005	0.031 ± 0.005
PTP duration (s)	0.010 ± 0.004	0.010 ± 0.005	0.011 ± 0.004
Twitch HRT (ms)	74.8 ± 9.4	72.0 ± 5.5	75.0 ± 6.1
Twitch CT (ms)	171.0 ± 12.8	168.6 ± 9.6	173.4 ± 7.5
Twitch average rise (N m/s)	0.035 ± 0.015	0.035 ± 0.016	0.036 ± 0.016
Twitch average fall (N m/s)	−0.023 ± 0.010	−0.023 ± 0.012	−0.024 ± 0.011

*Note*: All data are presented as means ± SD. Percentages relative to MVC torque are provided for the 40 Hz stimulated contractions. There was no significant difference between any baseline values (Table 1) across the three conditions (*P* = 0.08–0.95).

Abbreviations: CT, contraction time; HRT, half‐relaxation time; MVC, maximal voluntary contraction; PTP, peak‐to‐peak for M‐wave parameters.

### 3.2 Fatigue indices

There was a main effect of the fatiguing condition on the fatigue index (*P* < 0.001; Figure 2). *Post hoc* testing revealed that the 60 s exponentially decaying stimulation condition (40 → 20 Hz) demonstrated significantly less fatigue (i.e., higher fatigue index) than the constant 40 Hz high‐frequency and MVC conditions (Figure 2a; both *P* ≤ 0.001). There was no significant difference between the constant high‐frequency (40 Hz) and MVC conditions (*P* = 0.4, Figure 2a). For the MVC condition, VA was not significantly different at 55 s compared with baseline (97.6% ± 5.7% and 99.3% ± 1.3%, *P* = 0.33). Furthermore, during the MVC condition there was no effect for M‐wave parameters of area (70.5 ± 24.4 mV s, *P* = 0.5), peak‐to‐peak amplitude (6.7 ± 2.1 mV, *P* = 0.7), duration (0.031 ± 0.006 s, *P* = 0.9) and peak‐to‐peak duration (0.011 ± 0.006 s, *P* = 0.4) compared with baseline throughout the 60 s contraction.

### 3.3 Frequency switch

When the stimulation frequency was switched to 20 Hz for 2 s following the 60 s constant 40 Hz high‐frequency condition, torque increased significantly from 13.0 ± 4.0 to 15.2 ± 5.1 N m (16.9% increase, *P* < 0.005). When the stimulation frequency was switched to 40 Hz (from 20 Hz) for 2 s following the decaying stimulation condition (40 → 20 Hz), torque decreased significantly from 18.2 ± 5.1 to 15.3 ± 4.2 N m (15.9% decrease, *P* < 0.001), accompanied by a significant increase in the average rate of torque loss from −0.23 ± 0.14 to −1.44 ± 1.19 N m/s (*P* < 0.005). Exemplar torque data during the frequency switch are presented in Figure 3.

### 3.4 Task termination

At task termination (i.e., ∼2 s after the fatiguing condition), MVC torque was significantly depressed compared with baseline for all conditions (26.8 ± 7.5 N m, 27%–36% decrease, *P* < 0.001). The MVC at task termination for the decaying stimulation (40 → 20 Hz) condition was significantly lower than the MVC condition (9.6% ± 8.5% lower, *P* = 0.004), but not different from the constant high‐frequency condition (3.7% ± 11.5% lower, *P* = 0.27). There was no significant difference in MVC torque between the constant high‐frequency (40 Hz) and MVC condition (5.9% ± 11.3%, *P* = 0.16). For the constant high‐frequency condition (40 Hz), M‐wave peak‐to‐peak amplitude and area were significantly reduced (5.0 ± 2.8 mV and 45.5 ± 30.1 mV s, both ∼23% decrease) at task failure compared with baseline (*P* < 0.005), but duration and peak‐to‐peak duration remained unchanged relative to baseline (0.032 ± 0.006 and 0.011 ± 0.004 s, ∼1% and ∼13% increase, and *P* = 0.54 and *P* = 0.22, respectively). For the decaying stimulation (40 → 20 Hz) and MVC conditions at task termination, there was no significant difference in M‐wave peak‐to‐peak amplitude (6.5 ± 2.7 and 6.6 ± 2.0 mV, ∼8% and 0% decrease, and *P* = 0.08 and *P* = 0.91, respectively), area (66.7 ± 32.5 and 69.1 ± 21.8 mV s, ∼4% and ∼0% decrease, *P* = 0.46 and *P* = 0.95, respectively), duration (0.032 ± 0.005 and 0.031 ± 0.006 s, ∼1% and ∼0% increase, *P* = 0.39 and *P* = 0.89, respectively) or peak‐to‐peak duration (0.011 ± 0.004 and 0.012 ± 0.005 s, ∼12% and ∼16% increase, and *P* = 0.21 and *P* = 0.39, respectively) compared with baseline.

Compared with baseline, the constant high‐frequency (40 Hz) and decaying stimulation (40 → 20 Hz) conditions showed significant reductions in twitch torque (2.4 ± 1.2 and 2.5 ± 0.9 N m, ∼28% and ∼22% decrease, respectively, both *P* < 0.001) and average rate of rise (0.026 ± 0.015 and 0.025 ± 0.009 N m/s, ∼27% and ∼25% decrease, respectively, both *P* < 0.001), but increases in twitch contraction time (198.8 ± 24.6 and 226.7 ± 19.2 ms, ∼17% and ∼34% longer, both *P* < 0.01). Values of twitch torque and average rate of rise were not significantly different between the constant high‐frequency (40 Hz) and decaying stimulation (40 → 20 Hz) (*P* = 0.80 and *P* = 0.64, respectively), but the decaying stimulation condition (40 → 20 Hz) had a longer twitch contraction time than the constant high‐frequency (40 Hz) condition at task termination (*P* < 0.001). These twitch parameters were not significantly altered after the MVC condition (3.8 ± 1.6 N m, 0.038 ± 0.015 N m/s and 207.0 ± 28.5 ms, *P* = 0.37, *P* = 0.99 and *P* = 0.37, respectively). Twitch half‐relaxation time was significantly longer at task termination for the decaying stimulation condition (40 → 20 Hz) (127.2 ± 16.5 N m/s, ∼77% longer, *P* < 0.001), constant high‐frequency condition (40 Hz) (103.7 ± 21.7 N m/s, ∼41% longer, *P* < 0.005) and MVC condition (106.3 ± 16.3 N m/s, ∼30% longer, *P* < 0.001). The decaying stimulation condition (40 → 20 Hz) had a significantly slower twitch half‐relaxation time at task termination than the constant high‐frequency (40 Hz) (*P* < 0.001) and MVC conditions (*P* < 0.001). There was no significant difference in twitch half‐relaxation time between the constant high‐frequency (40 Hz) and MVC conditions (*P* = 0.68).

### 3.5 Recovery

At 2 min after task termination, M‐wave parameters recovered for the sustained 40 Hz stimulation condition (97%–107% baseline, *P* = 0.19–0.69) and were not significantly different from baseline values for the remainder of the 30 min recovery period (*P* = 0.72–0.99). All M‐wave parameters for the decaying simulation and MVC conditions were not altered at task termination (see above) and were also not significantly different from baseline at any other recovery time (95%–105% baseline, *P* = 0.77–0.99). For all conditions, MVC torque recovered to baseline values by 5 min after task termination (97%–98% baseline, *P* = 0.45–0.96).

The 10 Hz:40 Hz ratio at 30 min after task termination was significantly lower than baseline for the decaying stimulation condition (40 → 20 Hz) (0.25 ± 0.08, ∼26% lower, *P* < 0.001), constant high‐frequency condition (40 Hz) (0.29 ± 0.09, ∼17% lower, *P* < 0.001) and the MVC condition (0.28 ± 0.10, ∼26% lower, *P* < 0.001). The decaying stimulation condition (40 → 20 Hz) experienced a greater reduction in the 10 Hz:40 Hz ratio compared with the constant high‐frequency condition (40 Hz) (*P* = 0.006), but not the MVC condition (*P* = 0.12). There was no significant difference in the 10 Hz:40 Hz ratio between the constant high‐frequency condition (40 Hz) and MVC condition (*P* = 0.84).

## 4 DISCUSSION

Using electrically stimulated contractions, these data indicate support for the original muscle wisdom hypothesis by demonstrating that a reduction in physiological frequencies of activation preserves torque, rather than contributing to torque loss. Our results indicate that torque was better preserved because reducing rates of activation maintained both peripheral electrical propagation (i.e., M‐wave) and optimal muscle activation owing to peripheral slowing of contractile properties. Therefore, these findings from electrically stimulated contractions would indicate that the reduction in maximal MUFRs, well described for high‐intensity fatiguing contractions, does not contribute to torque loss, but instead is a compensatory mechanism that adapts to optimize force output as contractile properties slow. Despite what seems sensible functionally, it should be recognized that these concurrent changes in muscle properties and activation rates might be coincidental, and the relevance of their relationship is task dependent, as outlined below.

Although a similar methodological approach was used, our results contrast with those of Fuglevand and Keen (2003), who reported that sustaining physiological high rates of stimulation minimizes force loss, whereas reducing stimulation rates augments force loss. Using physiological rates of stimulation (30 Hz), which was an improvement on the design of the original experiments by proponents of muscle wisdom, in which they used high, unphysiological rates (80 Hz) to achieve high‐intensity forces (Bigland‐Ritchie et al., 1979; Jones et al., 1979; Marsden et al., 1983), Fuglevand and Keen (2003) were not able to induce a high‐intensity contraction, achieving only ∼55% of the MVC (Fuglevand & Keen, 2003). In the present study, we addressed both limitations from the prior studies by using physiological stimulation rates for the TA at an MVC of 40 Hz and were able to induce a high‐intensity contraction of ∼80% MVC with these rates (Table 1). This resulted in our findings being in agreement with the original studies (Bigland‐Ritchie et al., 1979; Jones et al., 1979; Marsden et al., 1983), which reported that force loss was mitigated by decaying rates of activation reflecting the decrease frequently reported in MUFRs during high‐intensity sustained contractions (Bigland‐Ritchie et al., 1983; Grimby & Hannerz, 1977; Marsden et al., 1983; cf. Macefield et al., 2000).

Task (defined here as the intensity of contraction) is well known to affect muscle force fatiguability during voluntary contractions. As mentioned, during higher intensities (i.e., ≥75% MVC), population recordings of MUFRs always decline (Bigland‐Ritchie et al., 1983; Dalton et al., 2010; Grimby & Hannerz, 1977; Marsden et al., 1983; cf. Macefield et al., 2000), but during submaximal intensities (≤50% MVC) the response of firing rates is variable (Garland et al., 1997) and might even increase (Carpentier et al., 2001; Valenčič et al., 2024) as a compensatory mechanism for force loss. Thus, at submaximal intensities the muscle wisdom hypothesis is not applicable (Garland & Gossen, 2002). The hypothesis was formulated with respect to sustained MVCs, and it is well appreciated that mechanisms for force loss and compensatory mechanisms that might mitigate this force loss differ depending on the task (Bigland‐Ritchie et al., 1995; Taylor & Gandevia, 2008); for example, during sustained MVCs the firing rates are presumably maximal and can only be maintained or decreased (Bigland‐Ritchie et al., 1983; Grimby & Hannerz, 1977). Conversely, during submaximal contractions an increase in firing rates can occur to compensate for torque impairments (Garland et al., 1997; Valenčič et al., 2024), a privilege that is unavailable during maximal contractions. In the study by Fuglevand and Keen (2003), electrically evoked twitches slowed after all three fatigue conditions, but as noted the condition with declining rates was most fatigable. Indeed, electrically evoked rates of muscle relaxation are not associated with MUFRs during sustained submaximal contractions (Garland et al., 1997), unlike sustained MVCs or high‐intensity contractions (i.e., ≥75% MVC) (Bigland‐Ritchie & Woods, 1984; Bigland‐Ritchie et al., 1983; Dalton et al., 2010). Therefore, owing to this task dependence, it is not surprising that maintenance of a high physiological frequency of stimulation demonstrated the lowest force loss in those experiments by Fuglevand and Keen (2003). With our present experiments addressing limitations from prior studies, it is clear that contraction intensity is a crucial variable for these interpretations.

The finding in the present study that reducing activation rates induced the least force loss (Figure 2) clearly is supported by modifying stimulation frequency immediately after the fatigue conditions. Specifically, torque output increased when 60 s of constant 40 Hz stimulation was immediately switched to 20 Hz for 2 s (i.e., 60–62 s; Figure 3b). Furthermore, immediately after 60 s of decaying stimulation (i.e., 40 → 20 Hz), increasing the frequency to 40 Hz accelerated torque loss (Figure 3c). These data are supportive of the muscle wisdom hypothesis and indicate that high physiological frequencies were suboptimal in maximizing torque production when the muscle was fatigued.

For optimal torque generation, activation rate should be well matched to the contractile speed of the muscle (Bigland‐Ritchie & Woods, 1984; Eccles et al., 1958; Kernell, 1995). Therefore, as contractile speed slows with fatigue, lower rates of activation are adequate to maintain optimal tetanic fusion (Bigland‐Ritchie & Woods, 1984; Bigland‐Ritchie et al., 1983). Indeed, following sustained muscle activation, fusion occurs at lower rates of activation (Bigland‐Ritchie & Woods, 1984; Davis & Davis, 1932) and the stimulated force–frequency relationship can shift leftwards owing to greater torque summation (Bigland‐Ritchie & Woods, 1984; cf. Binder‐Macleod & McDermond, 1992). In support, electrically evoked twitch half‐relaxation times slowed after all three fatigue conditions in the present study. Therefore, the decaying stimulation rate condition would match the contractile slowing and therefore provide optimal tetanic fusion. Consequently, during voluntary contractions this would maintain neural input to the necessary minimum for maximal torque output as peripheral contractile slowing occurs (i.e., muscle wisdom) (Figure 2 and 3).

In the present study, M‐waves were used to assess peripheral conduction. During sustained isometric MVCs, M‐wave amplitude has been shown to be unaffected (Bigland‐Ritchie et al., 1982; Merton, 1954); however, M‐wave duration often increases, presumably owing to slowing conduction velocity (Bigland‐Ritchie et al., 1979). Our results are comparable, because M‐wave amplitude did not decline during the sustained MVC condition; however, no increase of M‐wave duration was observed. Conversely, some studies have reported declining M‐wave amplitudes during sustained MVCs (Bellemare & Garzaniti, 1988; Stephens & Taylor, 1972); however, it is appreciated that these discrepancies might be primarily attributable to differences in the methodological approach and data analysis (see Bigland‐Ritchie et al., 1982; Thomas et al., 1995). Regardless, in our experimental model, all M‐wave parameters were unaffected during a sustained 1 min MVC, indicating that peripheral electrical conductance was maintained. When a high stimulation rate was sustained, there was a significant reduction in M‐wave amplitude at task termination. Conversely, there was no change in M‐wave amplitude for the decaying stimulation rate condition. Therefore, peripheral electrical propagation was maintained during high‐intensity activation only when stimulation rate declined.

The decrease in M‐wave amplitude is likely to reflect, in part, a compromise in the sarcolemmal action potential, and if decreases are large enough impairments in excitation contraction–coupling may occur, ultimately impairing torque output (Fuglevand, 1995). Thus, impaired electrical activation might contribute to the greater force loss during the sustained high‐frequency stimulation condition compared with the decaying condition, which maintains peripheral conduction (i.e., M‐wave). In contrast, Fuglevand and Keen (2003) reported no changes in M‐wave amplitude after 1 min of physiological high‐frequency stimulation. This discrepancy between studies is unclear but might be attributable to the muscle group tested because, for one example, differences in M‐wave parameters were observed during fatiguing conditions when comparing the TA with a hand muscle (Thomas et al., 1989). Furthermore, in support of our findings it has been reported that M‐wave amplitude declines during intermittent physiological high‐frequency (30 Hz) stimulation of the adductor pollicis muscle (Lanfranchi et al., 2024).

During recovery after fatiguing contractions, it is often observed that submaximal force‐generating capacity becomes impaired preferentially, referred to as prolonged low‐frequency force depression, which is characterized by a proportionally greater reduction of torque produced at lower frequencies (e.g., 10 Hz) compared with high frequencies of torque production (e.g., 40 Hz) (Allen et al., 2008). Here, we observed the presence of prolonged low‐frequency force depression following all conditions, but significantly more prolonged low‐frequency force depression for the decaying stimulation condition (40 → 20 Hz) in comparison to the constant high‐frequency (40 Hz) and MVC conditions. Observing greater prolonged low‐frequency force depression after the decaying stimulation was probably attributable to the greater fatigue resistance (i.e., less force loss) in this task and thus a greater time–torque integral, ultimately causing greater peripheral contractile impairment.

### 4.1 Considerations

Contrary to our hypothesis, the MVC condition produced a significantly lower fatigue index than the decaying stimulation condition. Despite the decaying stimulation being modelled after MUFRs during a sustained MVC, the greater absolute torque produced from the MVC could explain the greater fatigability observed in this condition (Bigland‐Ritchie et al., 1995).

Electrical stimulation was used to make inferences about voluntary contractions. However, supramaximal peripheral nerve stimulation induces synchronous activation of all motor units (MUs), which subsequently receive the same rate of activation; this does not occur under voluntary control. Furthermore, the firing rate profile of single MUs is diverse during sustained MVCs, and the rate of decline is variable across MUs (Grimby & Hannerz, 1977), in addition to differences in individual MU fatigability (Thomas et al., 1991). Artificial recruitment of all MUs was maintained during the electrically stimulated contractions; however, cessation of firing can be observed during voluntary contractions (Grimby & Hannerz, 1977; Peters & Fuglevand, 1999) and thus this aspect is not captured within the stimulation conditions. Importantly, these limitations are consistent with all studies contrasted here that led to opposing conclusions. Thus, we used the same methodology here to make comparisons. We were unable to match MVC torque completely, achieving ∼80% with high physiological rates of stimulation (Table 1); however, population recordings of single MUs during sustained contractions at 75% MVC also demonstrate significant reductions in MU firing rates (Dalton et al., 2010). In formulation of the muscle wisdom hypothesis, an overarching significance has been placed on contractile slowing (Bigland‐Ritchie & Woods, 1984; Marsden et al., 1983); however, other history‐dependent properties are important, which ultimately cause a force–frequency ‘hysteresis’ that might contribute to torque maintenance despite lowering rates of activation (Binder‐Macleod & Clamann, 1989).

## 5 CONCLUSION

Our findings indicate that a reduction in activation rate preserves torque, rather than contributing to torque loss during high‐intensity contractions, thereby supporting the muscle wisdom hypothesis. The reduction in stimulation rates is likely to have maintained optimal activation by matching the fatigue‐induced contractile slowing in combination with maintenance of peripheral electrical conductance. Therefore, these findings with electrically stimulated contractions support the hypothesis that the reduction in maximal MUFRs frequently reported from studies using high‐intensity voluntary contractions is an adaptive compensatory mechanism that helps to prevent torque loss.

## AUTHOR CONTRIBUTIONS

Raaj A. Dudani, Alexander M. Zero, Charles L. Rice all contributed to conception or design of the work, acquisition, analysis or interpretation of data for the work, and drafting the work or revising it critically for important intellectual content.

## CONFLICT OF INTEREST

The authors declare no conflicts of interest.

## Data Availability

Data available upon reasonable request.

## References

[eph13739-bib-0001] Allen, D. G. , Lamb, G. D. , & Westerblad, H. (2008). Skeletal muscle fatigue: Cellular mechanisms. Physiological Reviews, 88(1), 287–332. 18195089 10.1152/physrev.00015.2007

[eph13739-bib-0002] Barry, B. K. , & Enoka, R. M. (2007). The neurobiology of muscle fatigue: 15 years later. Integrative and Comparative Biology, 47(4), 465–473. 21672855 10.1093/icb/icm047

[eph13739-bib-0003] Bellemare, F. , & Garzaniti, N. (1988). Failure of neuromuscular propagation during human maximal voluntary contraction. Journal of Applied Physiology, 64(3), 1084–1093. 3366731 10.1152/jappl.1988.64.3.1084

[eph13739-bib-0004] Bellemare, F. , Woods, J. J. , Johansson, R. , & Bigland‐Ritchie, B. (1983). Motor‐unit discharge rates in maximal voluntary contractions of three human muscles. Journal of Neurophysiology, 50(6), 1380–1392. 6663333 10.1152/jn.1983.50.6.1380

[eph13739-bib-0005] Bigland‐Ritchie, B. , Johansson, R. , Lippold, J. C. J. J. , & Woods, J. B. (1983). Contractile speed and EMG changes during fatigue of sustained maximal voluntary contractions. Journal of Neurophysiology, 50(1), 313–324. 6308182 10.1152/jn.1983.50.1.313

[eph13739-bib-0006] Bigland‐Ritchie, B. , Johansson, R. , Lippold, O. C. , Smith, S. , & Woods, J. J. (1983). Changes in motoneurone firing rates during sustained maximal voluntary contractions. The Journal of Physiology, 340(1), 335–346. 6887053 10.1113/jphysiol.1983.sp014765 PMC1199212

[eph13739-bib-0007] Bigland‐Ritchie, B. , Jones, D. A. , & Woods, J. J. (1979). Excitation frequency and muscle fatigue: Electrical responses during human voluntary and stimulated contractions. Experimental Neurology, 64(2), 414–427. 428516 10.1016/0014-4886(79)90280-2

[eph13739-bib-0008] Bigland‐Ritchie, B. , Kukulka, C. G. , Lippold, O. C. , & Woods, J. J. (1982). The absence of neuromuscular transmission failure in sustained maximal voluntary contractions. The Journal of Physiology, 330(1), 265–278. 6294288 10.1113/jphysiol.1982.sp014340 PMC1225297

[eph13739-bib-0009] Bigland‐Ritchie, B. , Rice, C. L. , Garland, S. J. , & Walsh, M. L. (1995). Task‐dependent factors in fatigue of human voluntary contractions. Fatigue. Advances in Experimental Medicine and Biology, 384, 361–380. 8585465 10.1007/978-1-4899-1016-5_29

[eph13739-bib-0010] Bigland‐Ritchie, B. , & Woods, J. J. (1984). Changes in muscle contractile properties and neural control during human muscular fatigue. Muscle & Nerve, 7(9), 691–699. 6100456 10.1002/mus.880070902

[eph13739-bib-0011] Binder‐Macleod, S. A. , & Clamann, H. P. (1989). Force output of cat motor units stimulated with trains of linearly varying frequency. Journal of Neurophysiology, 61(1), 208–217. 2918346 10.1152/jn.1989.61.1.208

[eph13739-bib-0012] Binder‐Macleod, S. A. , & McDermond, L. R. (1992). Changes in the force‐frequency relationship of the human quadriceps femoris muscle following electrically and voluntarily induced fatigue. Physical Therapy, 72(2), 95–104. 1549641 10.1093/ptj/72.2.95

[eph13739-bib-0013] Carpentier, A. , Duchateau, J. , & Hainaut, K. (2001). Motor unit behaviour and contractile changes during fatigue in the human first dorsal interosseus. The Journal of Physiology, 534(3), 903–912. 11483719 10.1111/j.1469-7793.2001.00903.x PMC2278734

[eph13739-bib-0014] Connelly, D. M. , Rice, C. L. , Roos, M. R. , & Vandervoort, A. A. (1999). Motor unit firing rates and contractile properties in tibialis anterior of young and old men. Journal of Applied Physiology, 87(2), 843–852. 10444648 10.1152/jappl.1999.87.2.843

[eph13739-bib-0015] Dalton, B. H. , Harwood, B. , Davidson, A. W. , & Rice, C. L. (2010). Recovery of motoneuron output is delayed in old men following high‐intensity fatigue. Journal of Neurophysiology, 103(2), 977–985. 20032234 10.1152/jn.00908.2009

[eph13739-bib-0016] Davis, H. , & Davis, P. A. (1932). Fatigue in skeletal muscle in relation to the frequency of stimulation. American Journal of Physiology, 101(2), 339–356.

[eph13739-bib-0017] Eccles, J. C. , Eccles, R. M. , & Lundberg, A. (1958). The action potentials of the alpha motoneurones supplying fast and slow muscles. The Journal of Physiology, 142(2), 275–291. 13564435 10.1113/jphysiol.1958.sp006015 PMC1356679

[eph13739-bib-0018] Fuglevand, A. J. (1995). The role of the sarcolemma action potential in fatigue. In Fatigue. Advances in experimental medicine and biology (Vol. 384, pp. 101–108). Springer. 8585442 10.1007/978-1-4899-1016-5_8

[eph13739-bib-0019] Fuglevand, A. J. , & Keen, D. A. (2003). Re‐evaluation of muscle wisdom in the human adductor pollicis using physiological rates of stimulation. Journal of Physiology, 549(3), 865–875. 12717007 10.1113/jphysiol.2003.038836 PMC2342998

[eph13739-bib-0020] Garland, S. J. , & Gossen, E. R. (2002). The muscular wisdom hypothesis in human muscle fatigue. Exercise and Sport Sciences Reviews, 30(1), 45–49. 11800500 10.1097/00003677-200201000-00009

[eph13739-bib-0021] Garland, S. J. , Griffin, L. , & Ivanova, T. (1997). Motor unit discharge rate is not associated with muscle relaxation time in sustained submaximal contractions in humans. Neuroscience Letters, 239(1), 25–28. 9547163 10.1016/s0304-3940(97)00885-9

[eph13739-bib-0022] Grimby, L. , & Hannerz, J. (1977). Firing rate and recruitment order of toe extensor motor units in different modes of voluntary conraction. The Journal of Physiology, 264(3), 865–879. 845828 10.1113/jphysiol.1977.sp011699 PMC1307796

[eph13739-bib-0023] Jones, D. A. , Bigland‐Ritchie, B. , & Edwards’, R. H. T. (1979). Excitation frequency and muscle fatigue: Mechanical responses during voluntary and stimulated contractions. Experimental Neurology, 64(2), 401–413. 428515 10.1016/0014-4886(79)90279-6

[eph13739-bib-0024] Kernell, D. (1995). Neuromuscular frequency‐coding and fatigue. In Fatigue. Advances in experimental medicine and biology (Vol. 384, pp. 135–145). Springer. 8585446 10.1007/978-1-4899-1016-5_11

[eph13739-bib-0025] Lanfranchi, C. , Rodriguez‐Falces, J. , & Place, N. (2024). The first and second phases of the muscle compound action potential in the thumb are differently affected by electrical stimulation trains. Journal of Applied Physiology, 136(5), 1122–1128. 38511213 10.1152/japplphysiol.00861.2023

[eph13739-bib-0026] Macefield, V. G. , Fuglevand, A. J. , Howell, J. N. , & Bigland‐Ritchie, B. (2000). Discharge behaviour of single motor units during maximal voluntary contractions of a human toe extensor. The Journal of Physiology, 528(1), 227–234. 11018121 10.1111/j.1469-7793.2000.00227.x PMC2270114

[eph13739-bib-0027] Marsden, C. D. , Meadows, J. C. , & Merton, P. A. (1976). Proceedings: Fatigue in human muscle in relation to the number and frequency of motor impulses. The Journal of Physiology, 258(2), 94P–95P. 957199

[eph13739-bib-0028] Marsden, C. D. , Meadows, J. C. , & Merton, P. A. (1983). “Muscular wisdom” that minimizes fatigue during prolonged effort in man: Peak rates of motoneuron discharge and slowing of discharge during fatigue. Motor Control Mechanisms in Health and Disease, 39, 169–211. 6229158

[eph13739-bib-0029] Marsh, E. , Sale, D. , McComas, A. J. , Quinlan, J. , & McComas, A. J. (1981). Influence of joint position on ankle dorsiflexion in humans. Journal of Applied Physiology: Respiratory, Environmental and Exercise Physiology, 51(1), 160–167. 7263411 10.1152/jappl.1981.51.1.160

[eph13739-bib-0030] Merton, P. A. (1954). Voluntary strength and fatigue. The Journal of Physiology, 123(3), 553–564. 13152698 10.1113/jphysiol.1954.sp005070 PMC1366225

[eph13739-bib-0031] Peters, E. J. D. , & Fuglevand, A. J. (1999). Cessation of human motor unit discharge during sustained maximal voluntary contraction. Neuroscience Letters, 274(1), 66–70. 10530521 10.1016/s0304-3940(99)00666-7

[eph13739-bib-0032] Power, G. A. , Dalton, B. H. , Behm, D. G. , Vandervoort, A. A. , Doherty, T. J. , & Rice, C. L. (2010). Motor unit number estimates in masters runners: Use it or lose it? Medicine and Science in Sports and Exercise, 42(9), 1644–1650. 20142771 10.1249/MSS.0b013e3181d6f9e9

[eph13739-bib-0033] Smith, C. B. , Allen, M. D. , & Rice, C. L. (2014). Voluntary rate of torque development is impaired after a voluntary versus tetanic conditioning contraction. Muscle and Nerve, 49(2), 218–224. 23625611 10.1002/mus.23888

[eph13739-bib-0034] Stephens, J. A. , & Taylor, A. (1972). Fatigue of maintained voluntary muscle contraction in man. The Journal of physiology, 220(1), 1–18. 5059236 10.1113/jphysiol.1972.sp009691 PMC1331686

[eph13739-bib-0035] Taylor, J. L. , Amann, M. , Duchateau, J. , Meeusen, R. , & Rice, C. L. (2016). Neural contributions to muscle fatigue: From the brain to the muscle and back again. Medicine and Science in Sports and Exercise, 48(11), 2294–2306. 27003703 10.1249/MSS.0000000000000923 PMC5033663

[eph13739-bib-0036] Taylor, J. L. , & Gandevia, S. C. (2008). A comparison of central aspects of fatigue in submaximal and maximal voluntary contractions. Journal of Applied Physiology, 104(2), 542–550. 18032577 10.1152/japplphysiol.01053.2007

[eph13739-bib-0037] Thomas, C. K. , Enoka, R. M. , Gandevia, S. C. , McComas, A. J. , & Stuart, D. G. (1995). The scientific contributions of brenda bigland‐ritchie. In Fatigue. Advances in experimental medicine and biology (Vol. 384, pp. 11–25). Springer. 8585444 10.1007/978-1-4899-1016-5_2

[eph13739-bib-0038] Thomas, C. K. , Johansson, R. S. , & Bigland‐Ritchie, B. (1991). Attempts to physiologically classify human thenar motor units. Journal of Neurophysiology, 65(6), 1501–1508. 1875258 10.1152/jn.1991.65.6.1501

[eph13739-bib-0039] Thomas, C. K. , Woods, J. J. , & Bigland‐Ritchie, B. (1989). Impulse propagation and muscle activation in long maximal voluntary contractions. Journal of Applied Physiology, 67(5), 1835–1842. 2557321 10.1152/jappl.1989.67.5.1835

[eph13739-bib-0040] Todd, G. , Gorman, R. B. , & Gandevia, S. C. (2004). Measurement and reproducibility of strength and voluntary activation of lower‐limb muscles. Muscle & Nerve, 29(6), 834–842. 15170616 10.1002/mus.20027

[eph13739-bib-0041] Valenčič, T. , Ansdell, P. , Brownstein, C. G. , Spillane, P. M. , Holobar, A. , & Škarabot, J. (2024). Motor unit discharge rate modulation during isometric contractions to failure is intensity‐ and modality‐dependent. The Journal of Physiology, 602(10), 2287–2314. 38619366 10.1113/JP286143

[eph13739-bib-0042] Zero, A. M. , Fanous, J. , & Rice, C. L. (2023). Acute and prolonged competing effects of activation history on human motor unit firing rates during contractile impairment and recovery. The Journal of Physiology, 601(24), 5689–5703. 37962903 10.1113/JP285189

